# Supplementary material for: The N’-Substituted Derivatives of 5-Chloro-3-Methylisothiazole-4-Carboxylic Acid Hydrazide with Antiproliferative Activity
Source: Molecules. 2019 Dec 25;25(1):88. doi: 10.3390/molecules25010088 (PMC6982951; doi:10.3390/molecules25010088)

Supplementary Material

The *N′*-Substituted Derivatives of 5-Chloro-3-Methylisothiazole-4-Carboxylic Acid Hydrazide with Antiproliferative Activity

Izabela Jęśkowiak [1,*], Stanisław Ryng [1], Marta Świtalska [2], Joanna Wietrzyk [2], Iwona Bryndal [3], Tadeusz Lis [4] and Marcin Mączyński [1]

[1] Department of Organic Chemistry, Faculty of Pharmacy, Wrocław Medical University, 211A Borowska Str, 50-556 Wrocław, Poland; stanislaw.ryng@umed.wroc.pl (S.R.); marcin.maczynski@umed.wroc.pl (M.M.)
[2] Institute of Immunology and Experimental Therapy, Polish Academy of Sciences, R. Weigla 12, 53-114 Wrocław, Poland; marta.switalska@hirszfeld.pl (M.Ś.); joanna.wietrzyk@hirszfeld.pl (J.W.)
[3] Department of Drug Technology, Faculty of Pharmacy, Wrocław Medical University, 211A Borowska Str, 50-556 Wrocław, Poland; iwona.bryndal@umed.wroc.pl
[4] Faculty of Chemistry, University of Wrocław, 14 Joliot-Curie, 50-383 Wrocław, Poland; tadeusz.lis@chem.uni.wroc.pl
* Correspondence: izabela.jeskowiak@student.umed.wroc.pl

Received: 4 December 2019; Accepted: 22 December 2019; Published: 24 December 2019

Figure S1. ESI-MS spectrum of compound **2.**

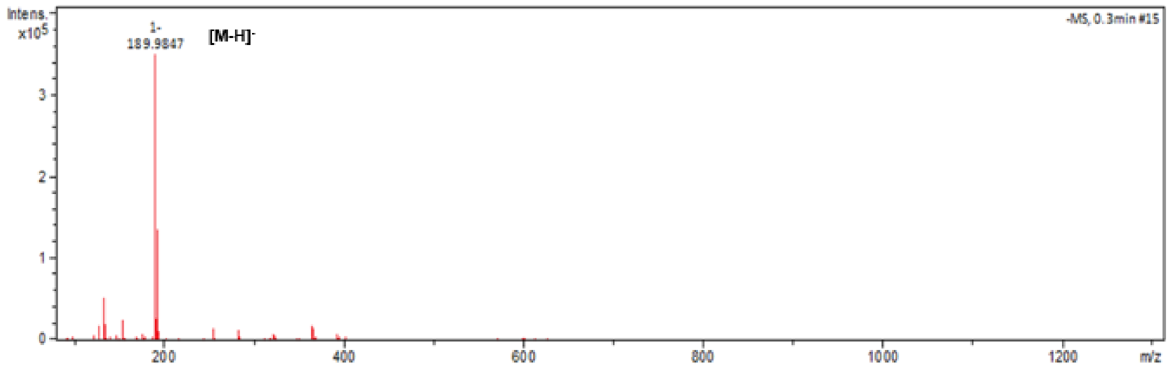

Figure S2. ESI-MS spectrum of compound **3.**

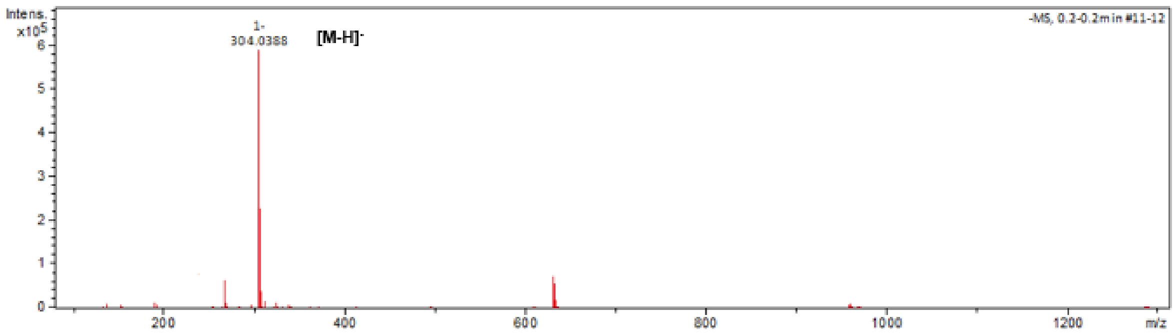

Figure S3. ESI-MS spectrum of compound **4.**

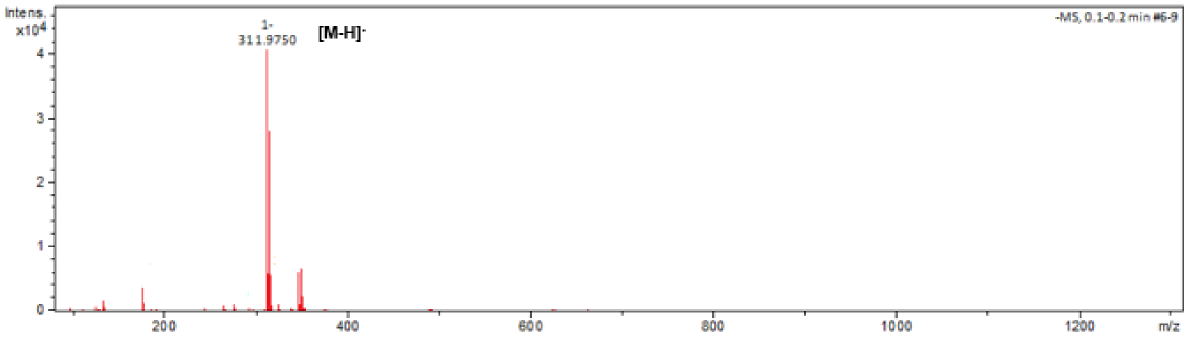
Intens.
x10^4
1-
311.9750
[M-H]-
-MS, 0.1-0.2 min #6-9
m/z

Figure S4. ESI-MS spectrum of compound **5.**

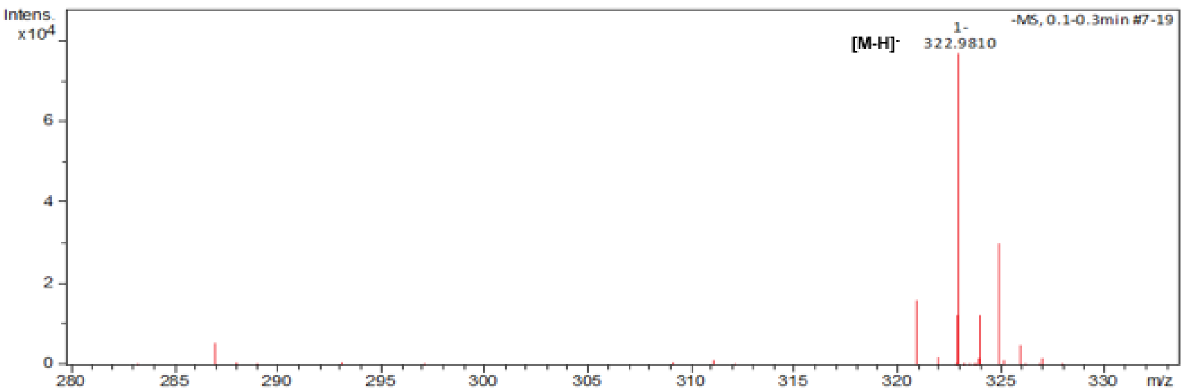

Figure S5. ESI-MS spectrum of compound **6.**

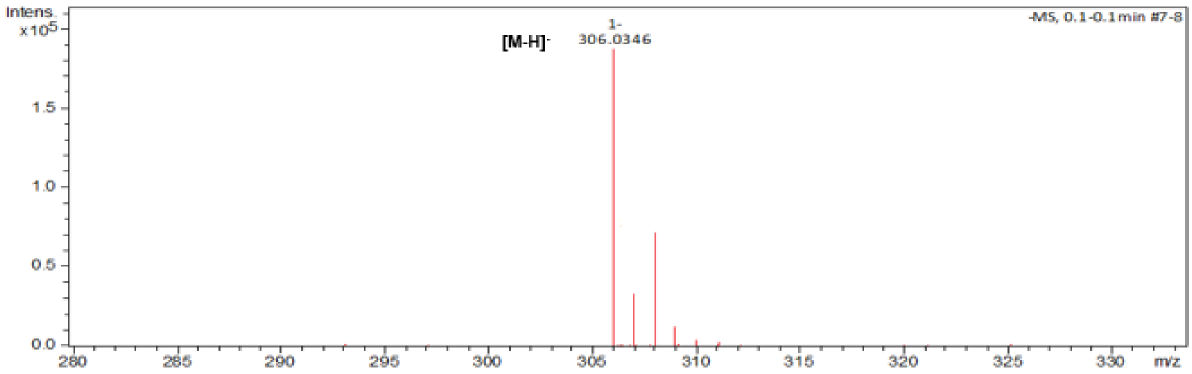

Figure S6. ESI-MS spectrum of compound **7.**

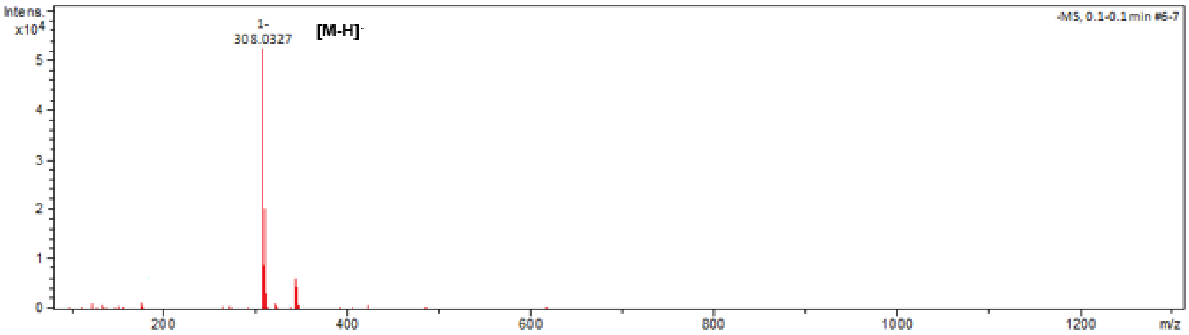

Figure S7. ESI-MS spectrum of compound **8.**

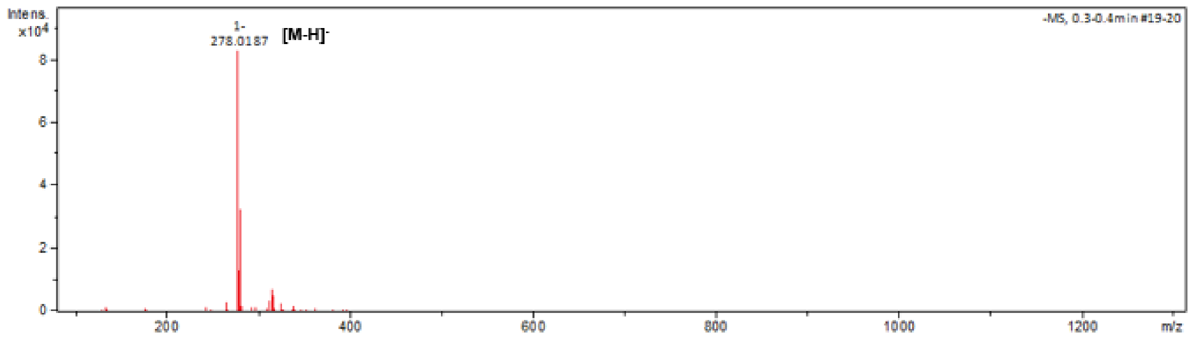

Figure S8. ESI-MS spectrum of compound **9.**

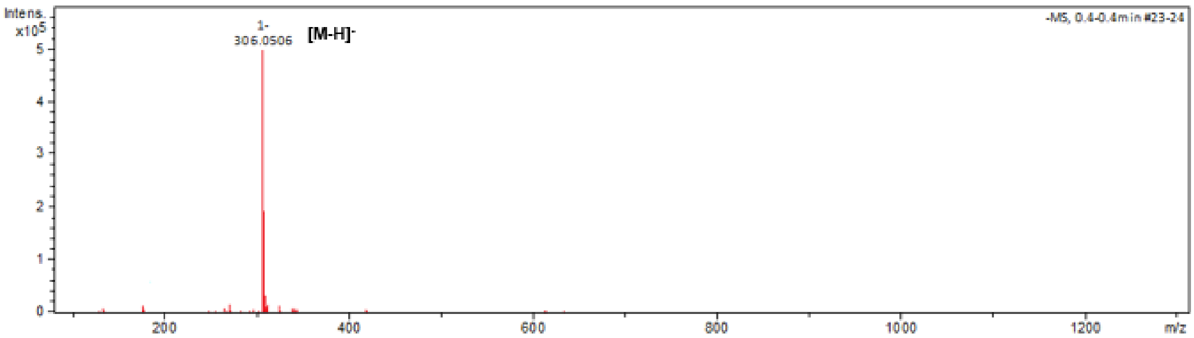

Figure S9. ESI-MS spectrum of compound **10.**

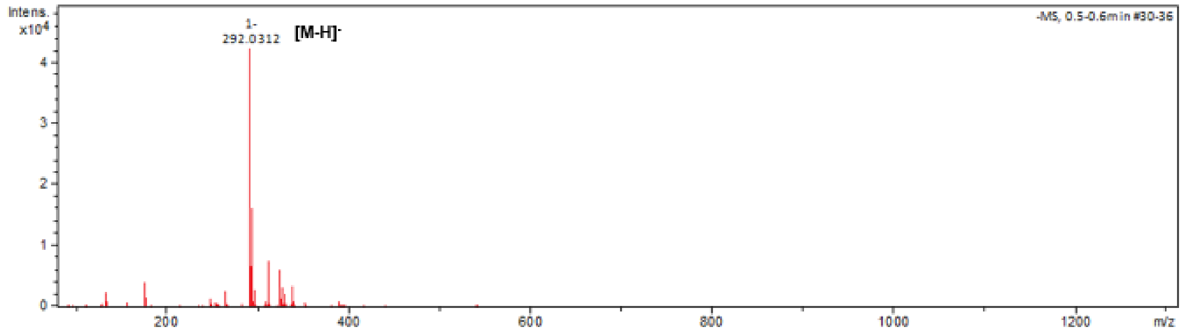

Figure S10. ESI-MS spectrum of compound **11.**

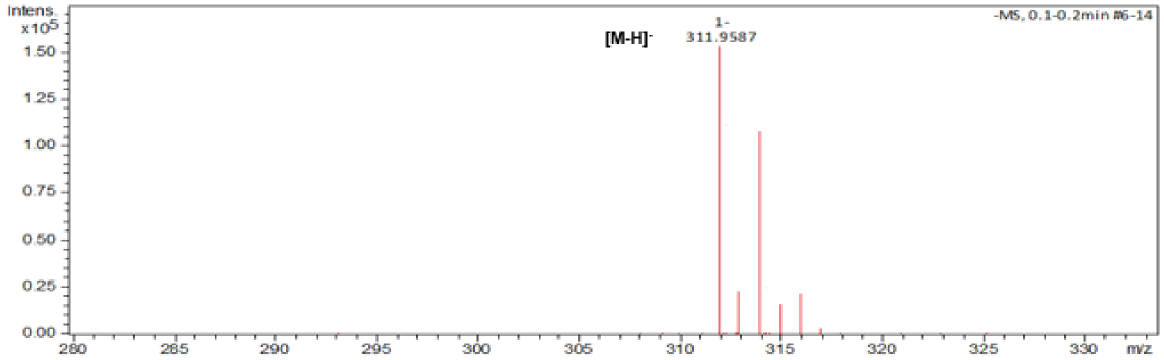

Figure S11. ^{1}H NMR spectrum of compound **2.**

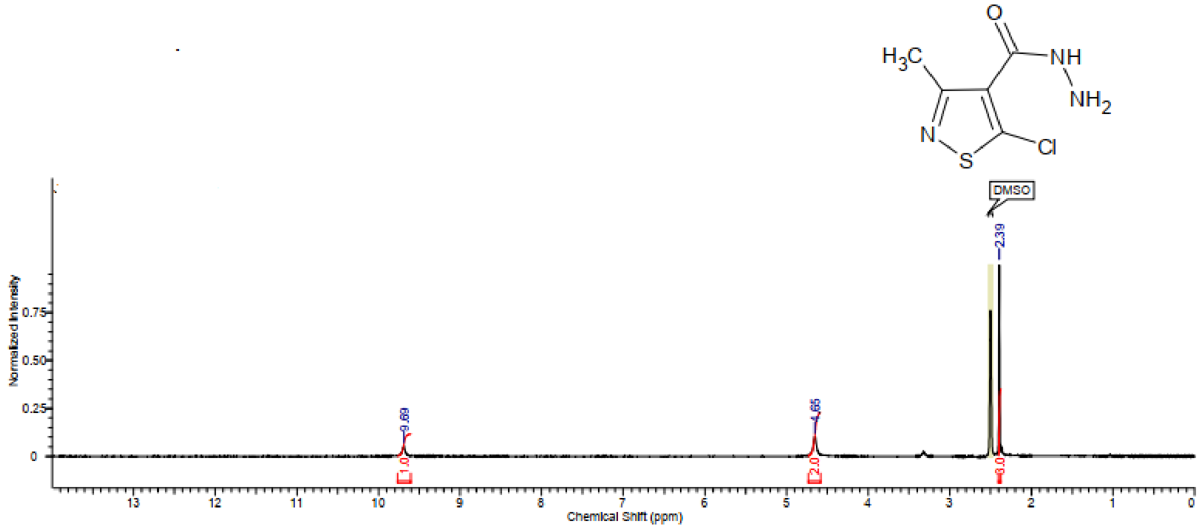

Figure S12. ^{1}H NMR spectrum of compound **3.**

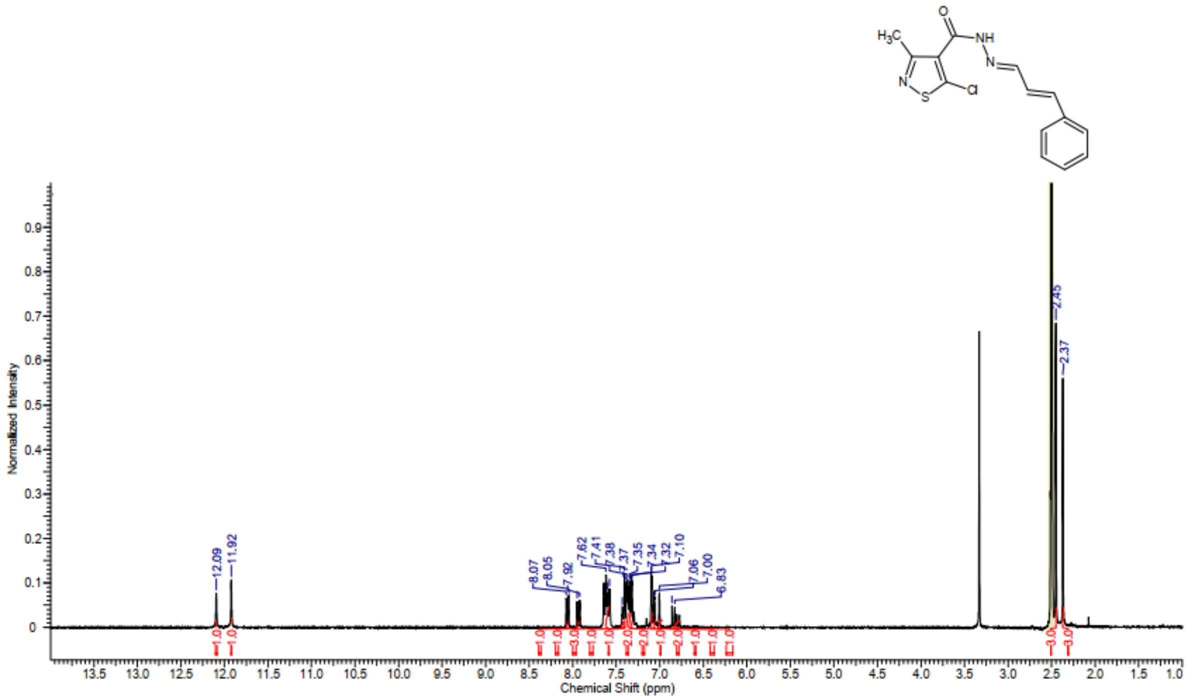

Figure S13. ^{1}H NMR spectrum of compound **4.**

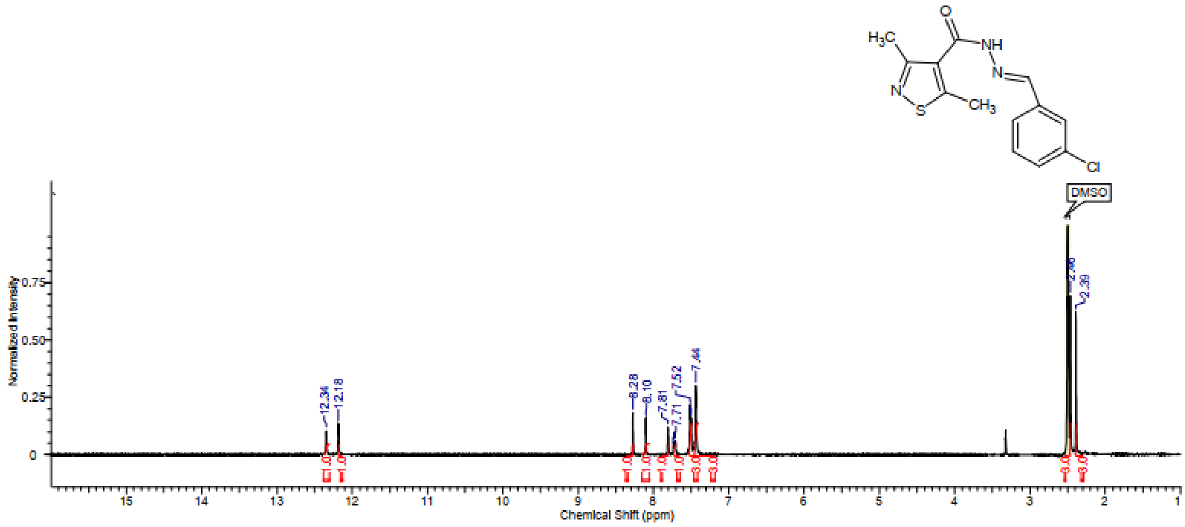

Figure S14. ^{1}H NMR spectrum of compound **5.**

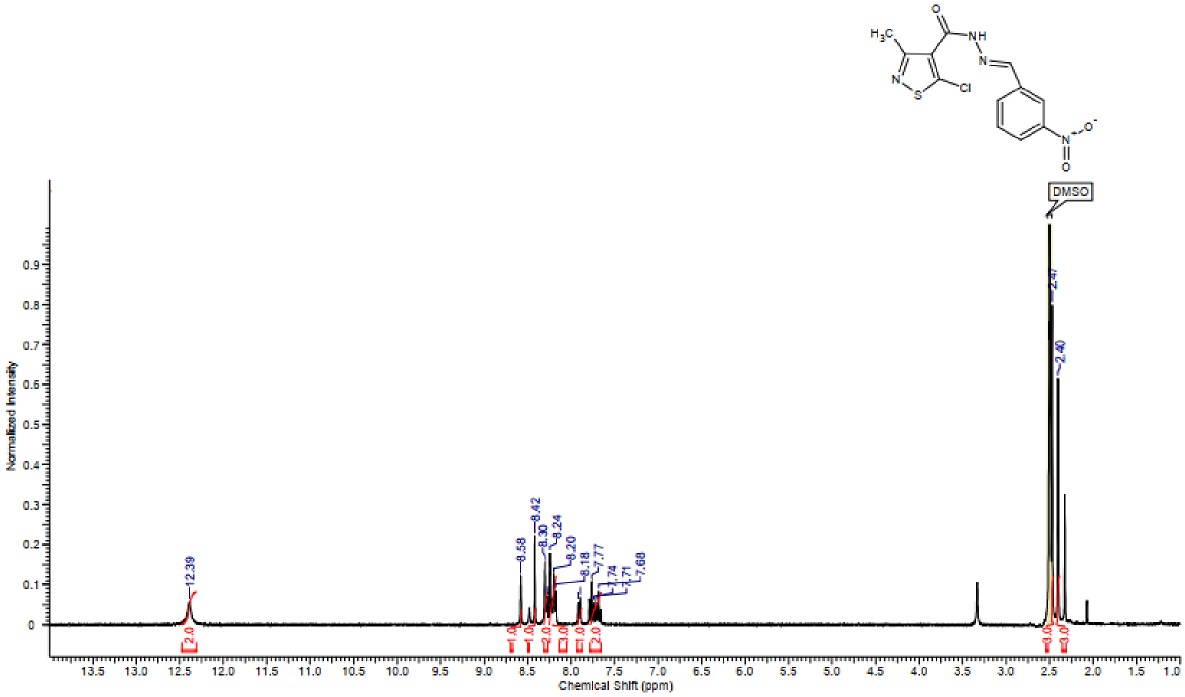

Figure S15. ^{1}H NMR spectrum of compound **6.**

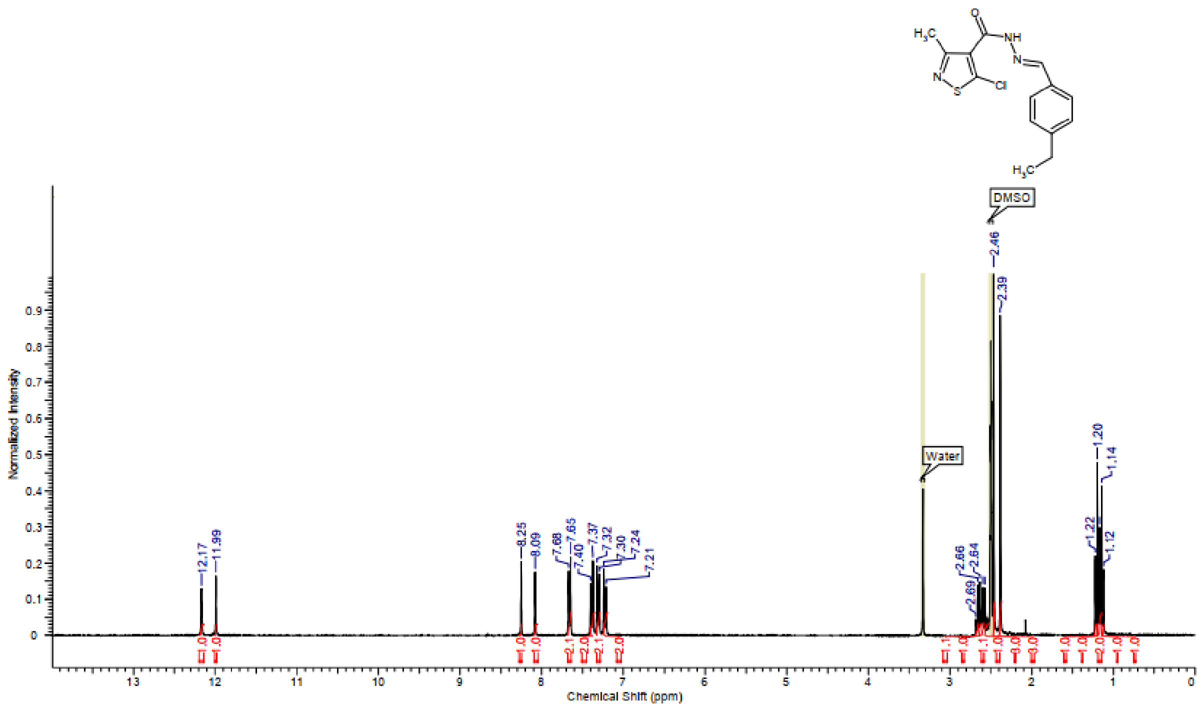

Figure S16. ^{1}H MR spectrum of compound **7.**

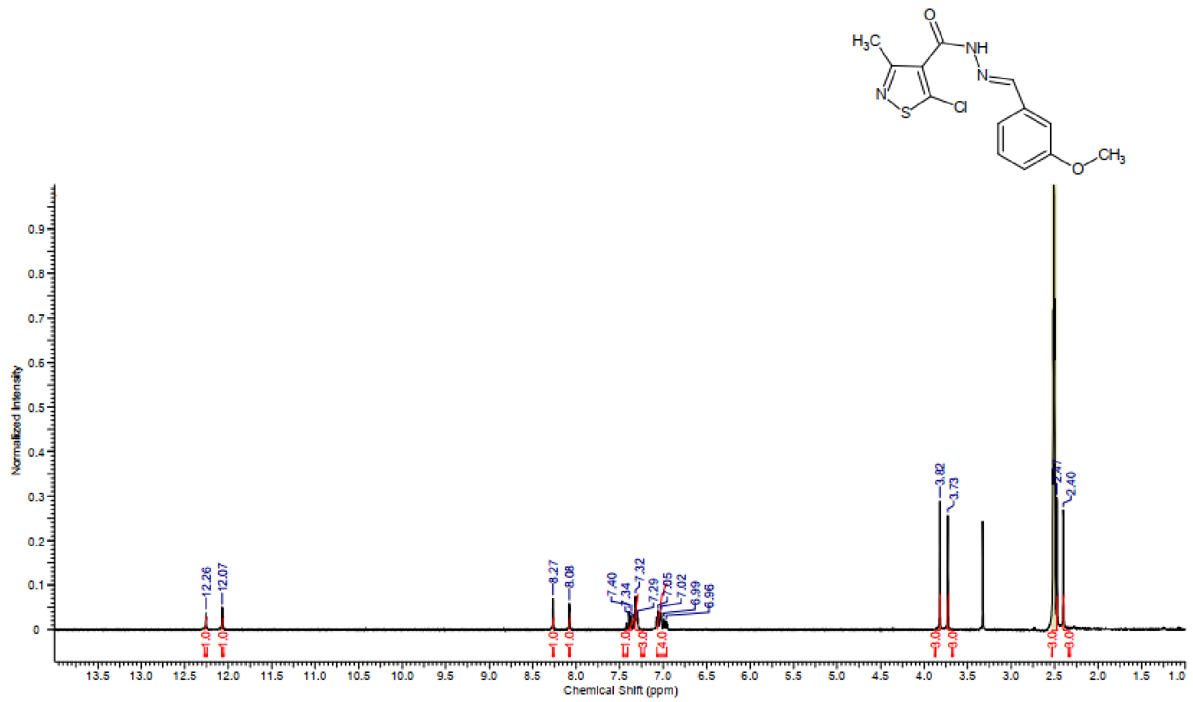

Figure S17. ¹H NMR spectrum of compound **8.**

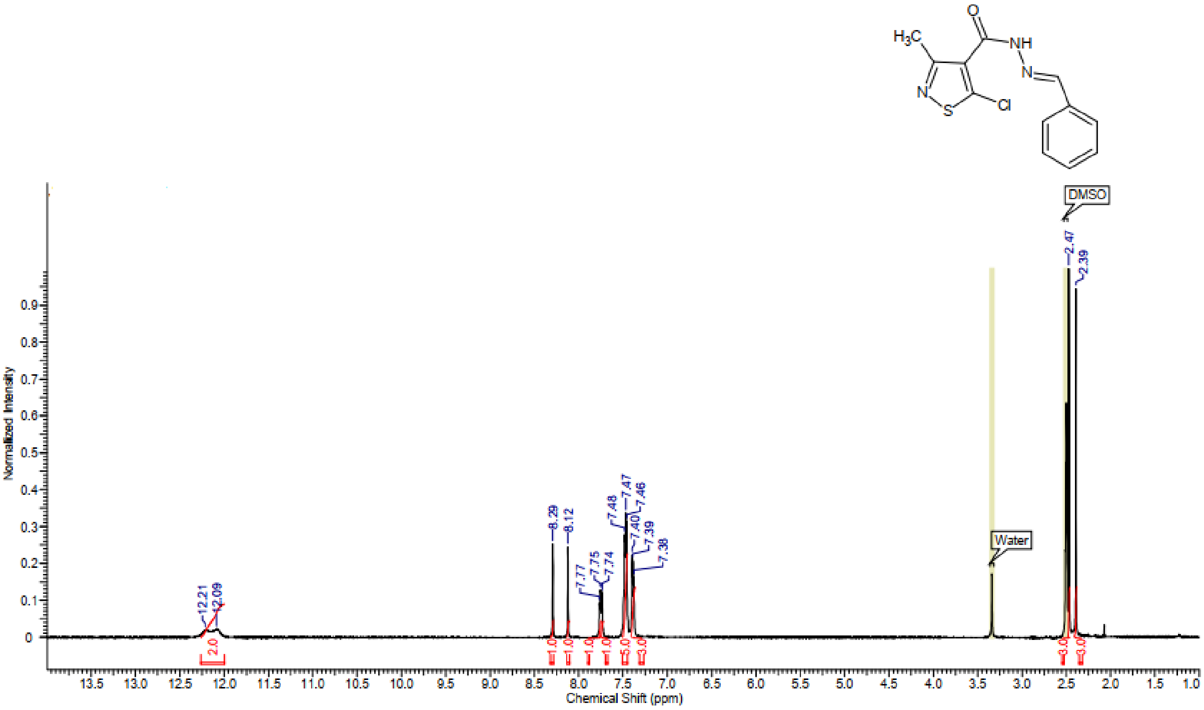

Figure S18. ¹H NMR spectrum of compound **9.**

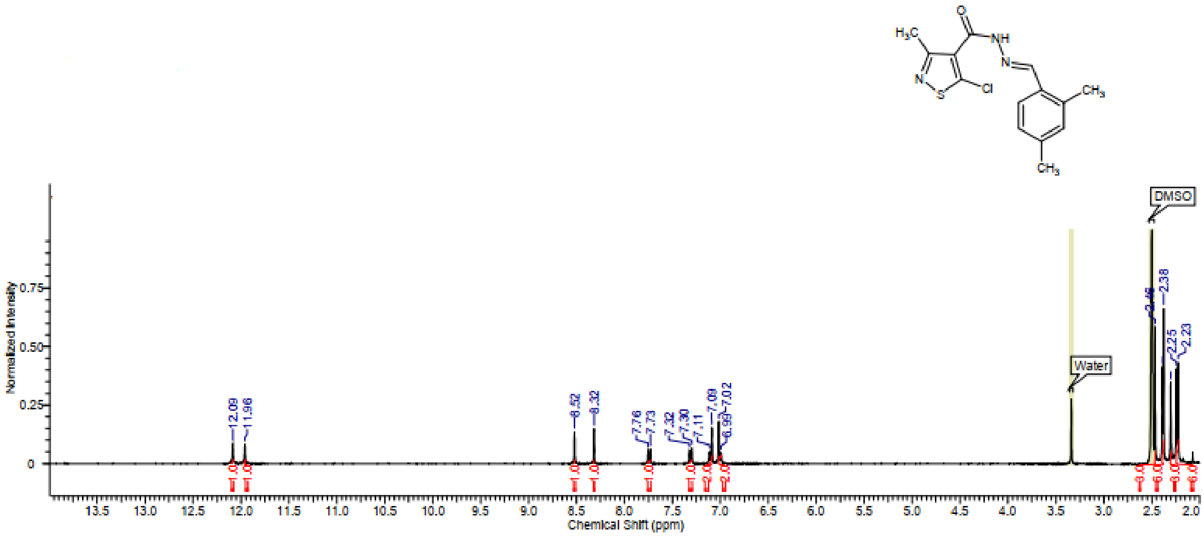

Figure S19. ^{1}H NMR spectrum of compound **10.**

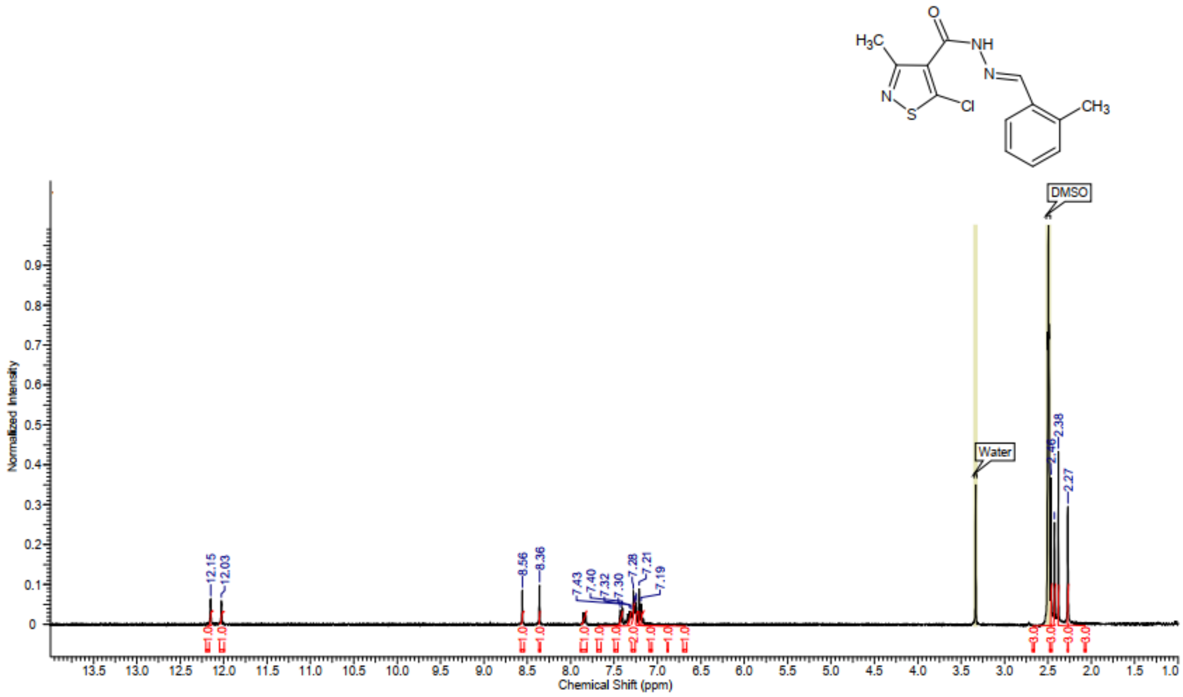

Figure S20. ^{1}H NMR spectrum of compound **11.**

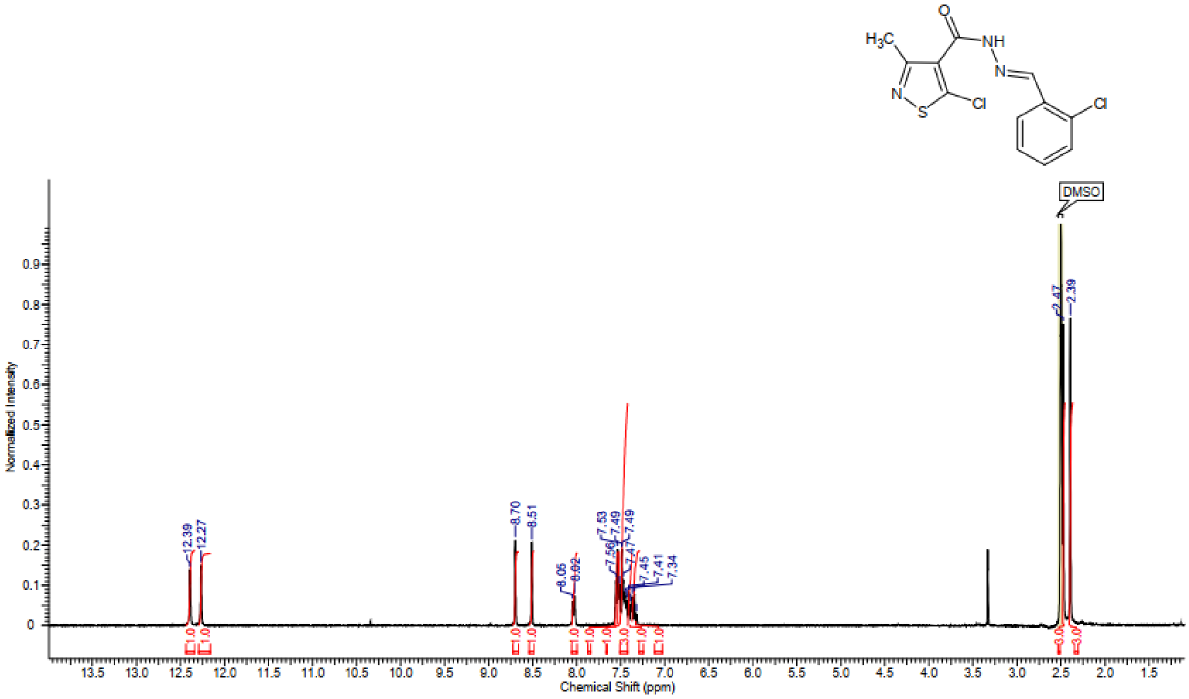

Figure S21. ^{13}C NMR spectrum of compound **2.**

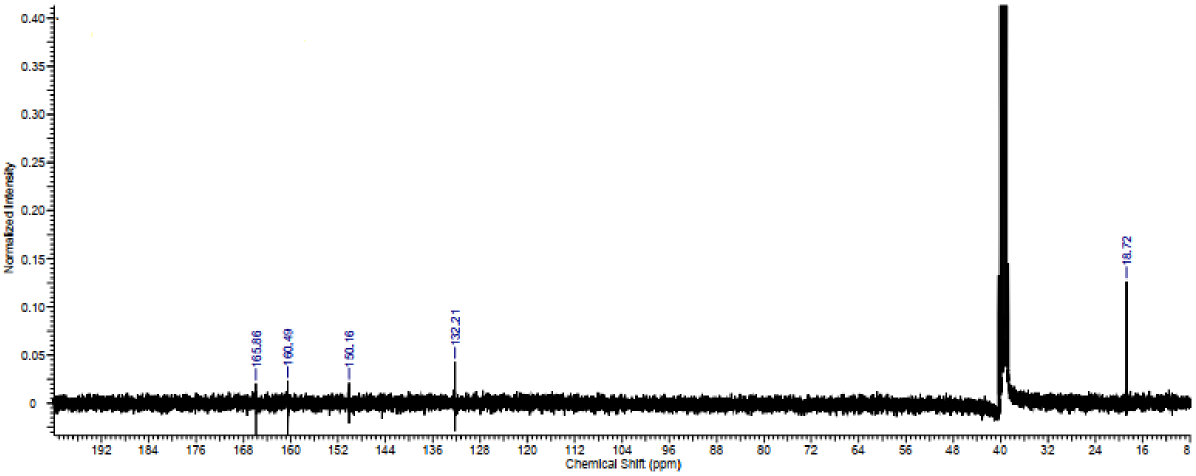

Figure S22. ^{13}C NMR spectrum of compound **3.**

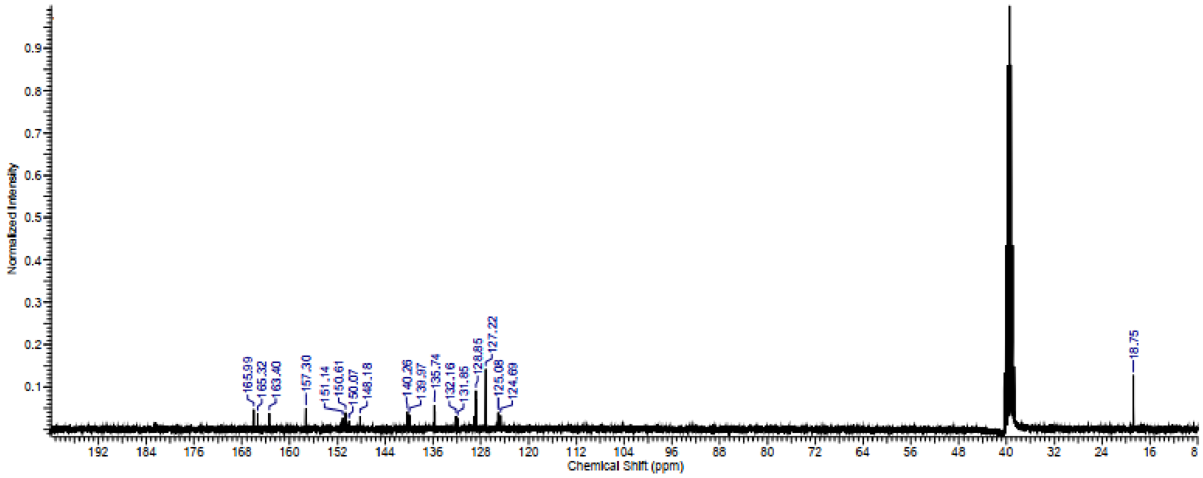

Figure S23. ^{13}C NMR spectrum of compound **4.**

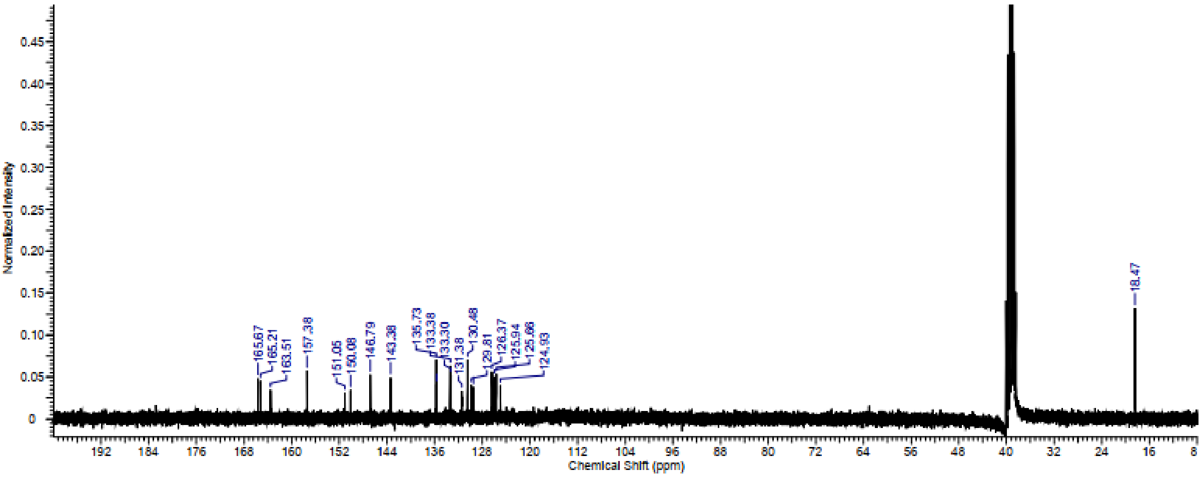

Figure S24. ^{13}C NMR spectrum of compound **5.**

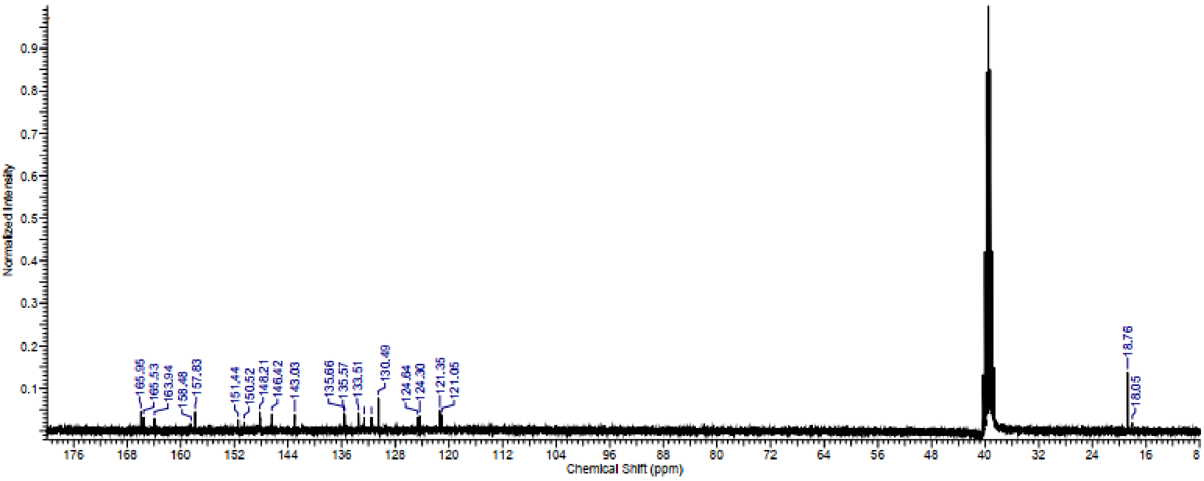

Figure S25. ^{13}C NMR spectrum of compound **6.**

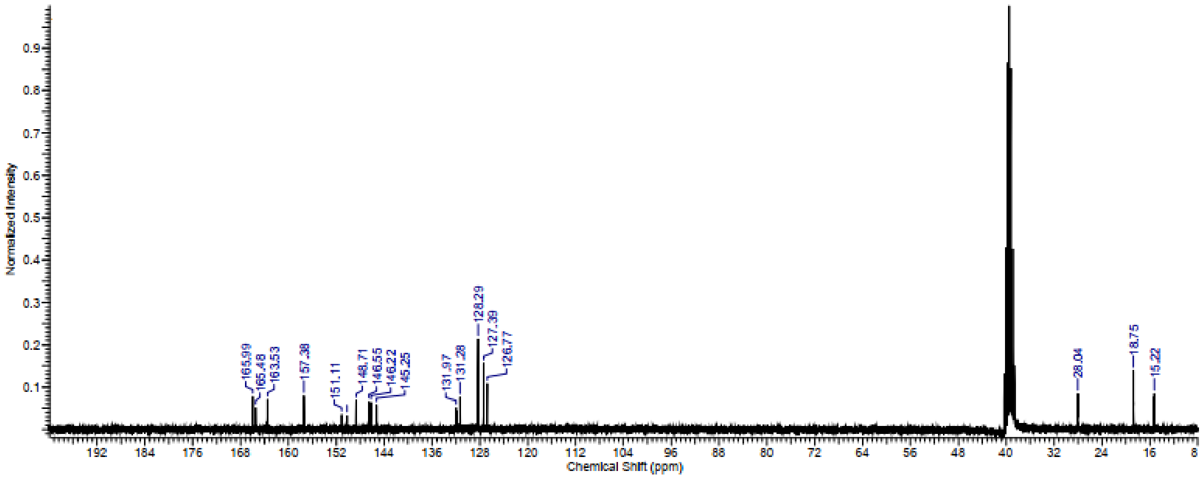

Figure S26. ^{13}C NMR spectrum of compound **7.**

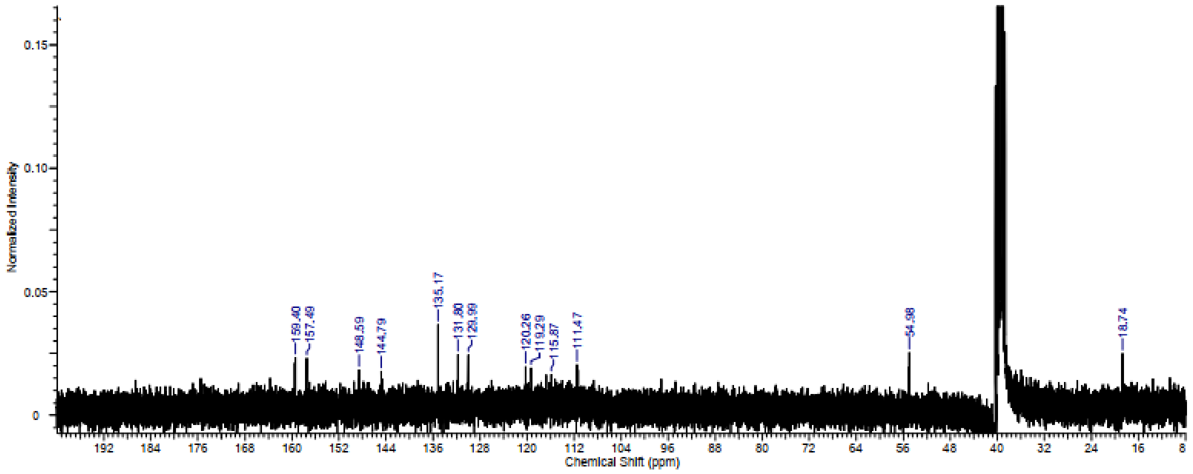

Figure S27. ^{13}C NMR spectrum of compound **8.**

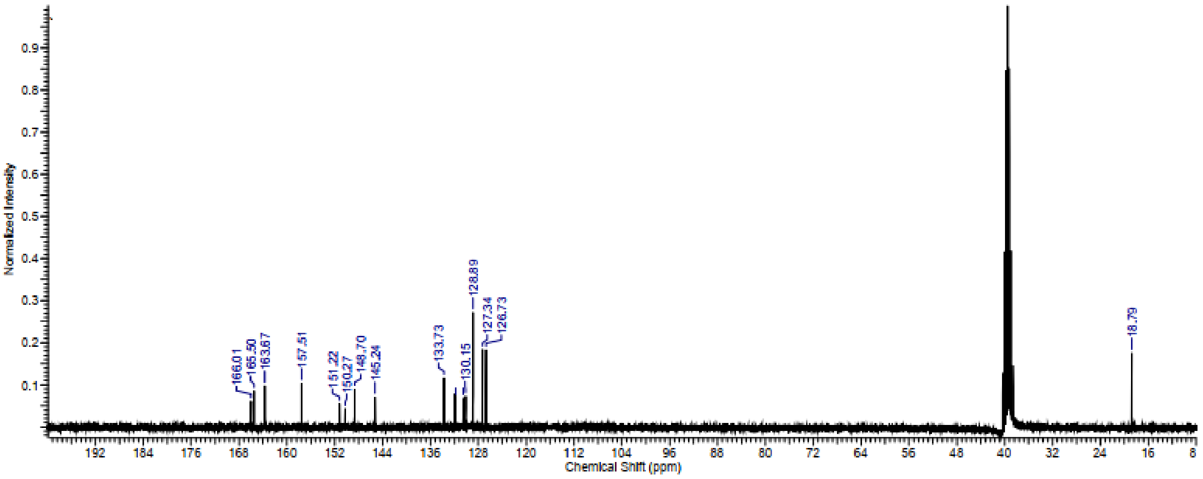

Figure S28. ^{13}C NMR spectrum of compound **9.**

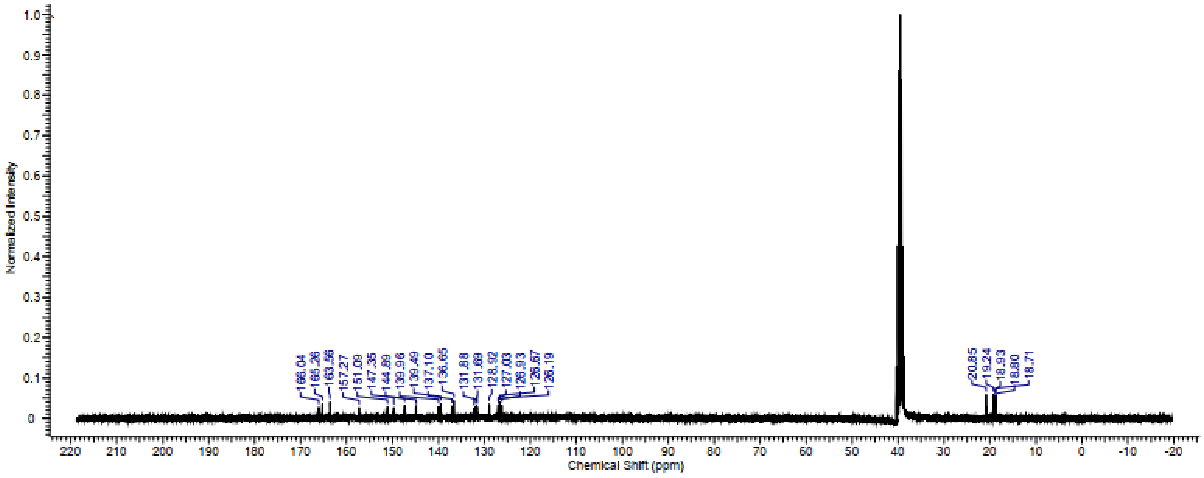

Figure S29. ^{13}C NMR spectrum of compound **10.**

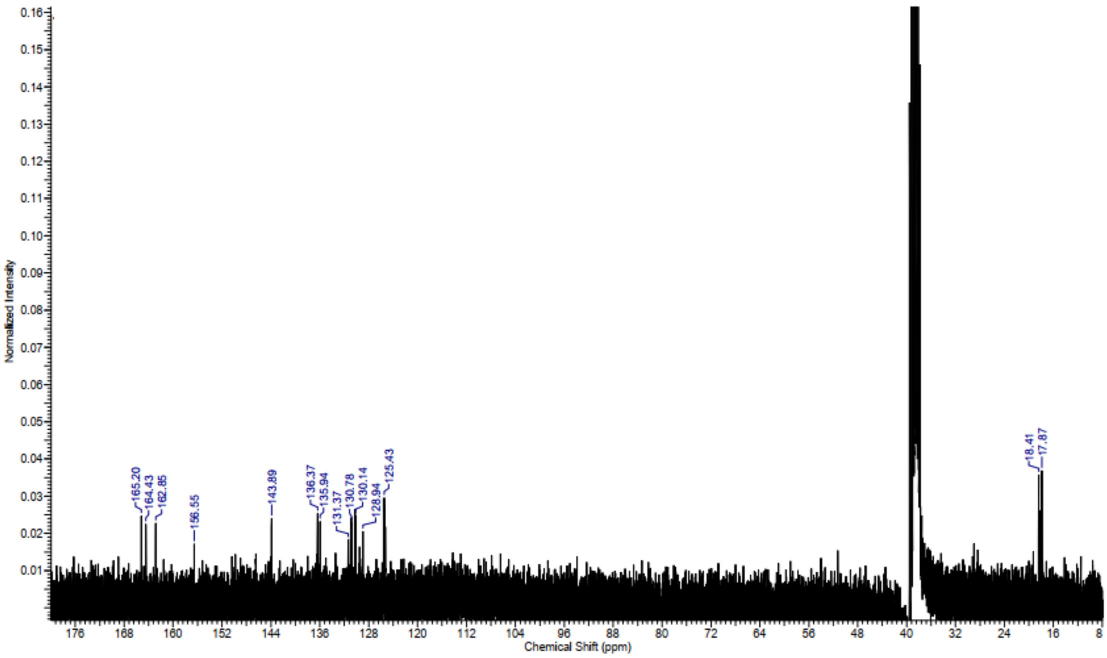

Figure S30. ^{13}C NMR spectrum of compound **11.**

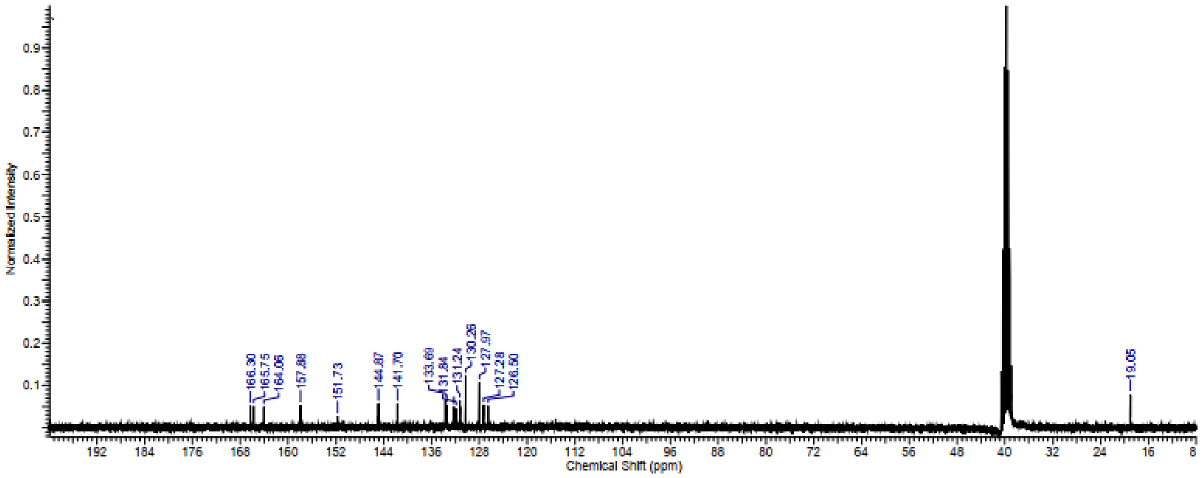

Figure S31. ^{1}H-^{13}C NMR spectrum of compound **2.**

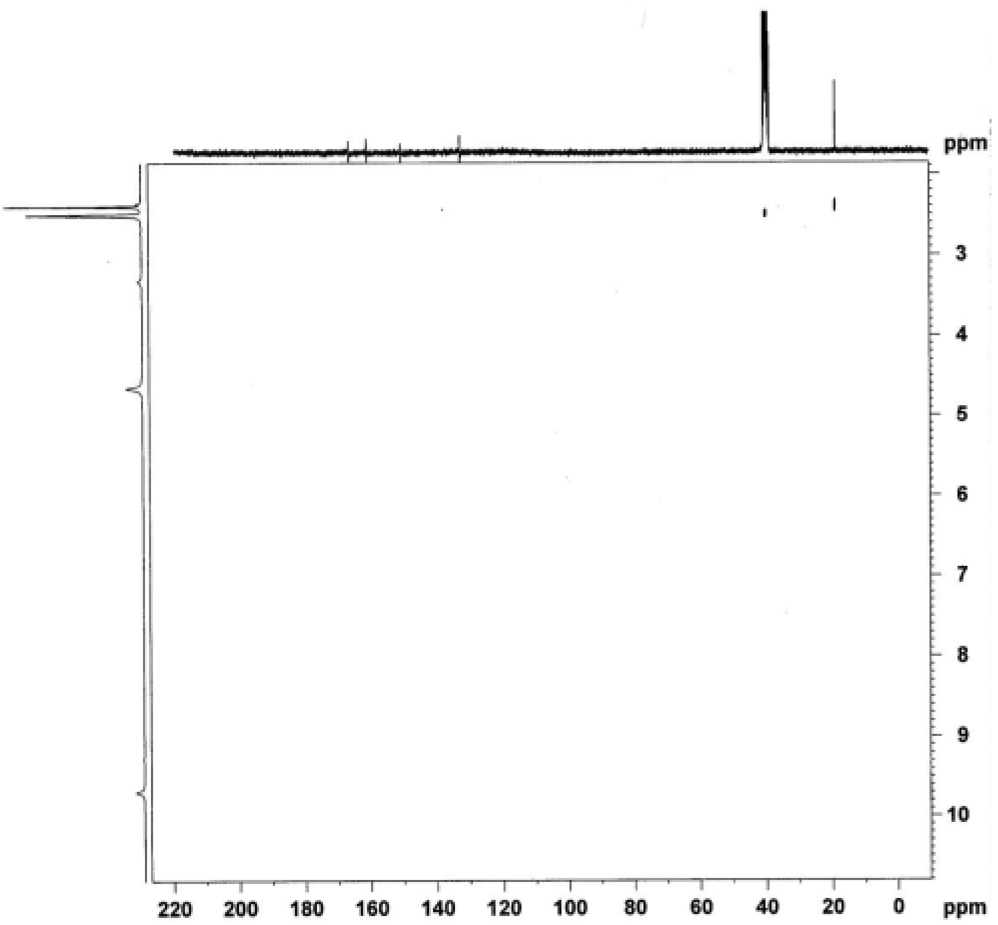

Figure S32. ^1H-^{13}C NMR spectrum of compound **3.**

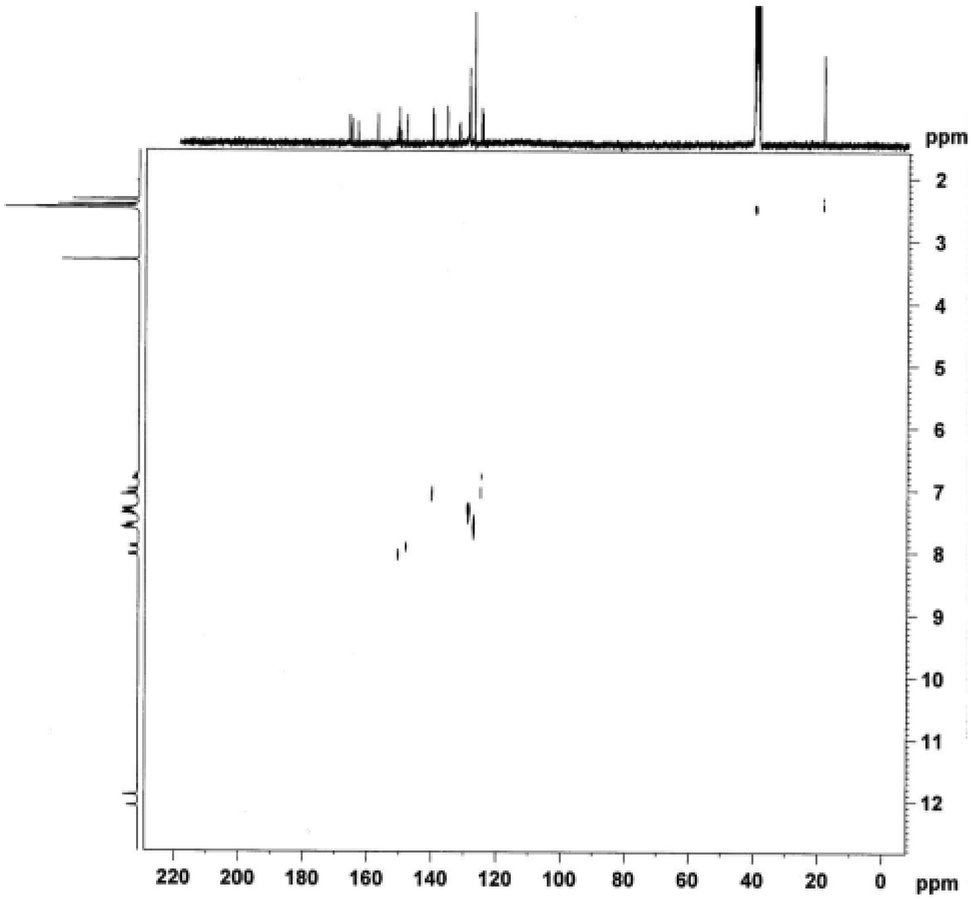

Figure 33. ^{1}H-^{13}C NMR spectrum of compound **4.**

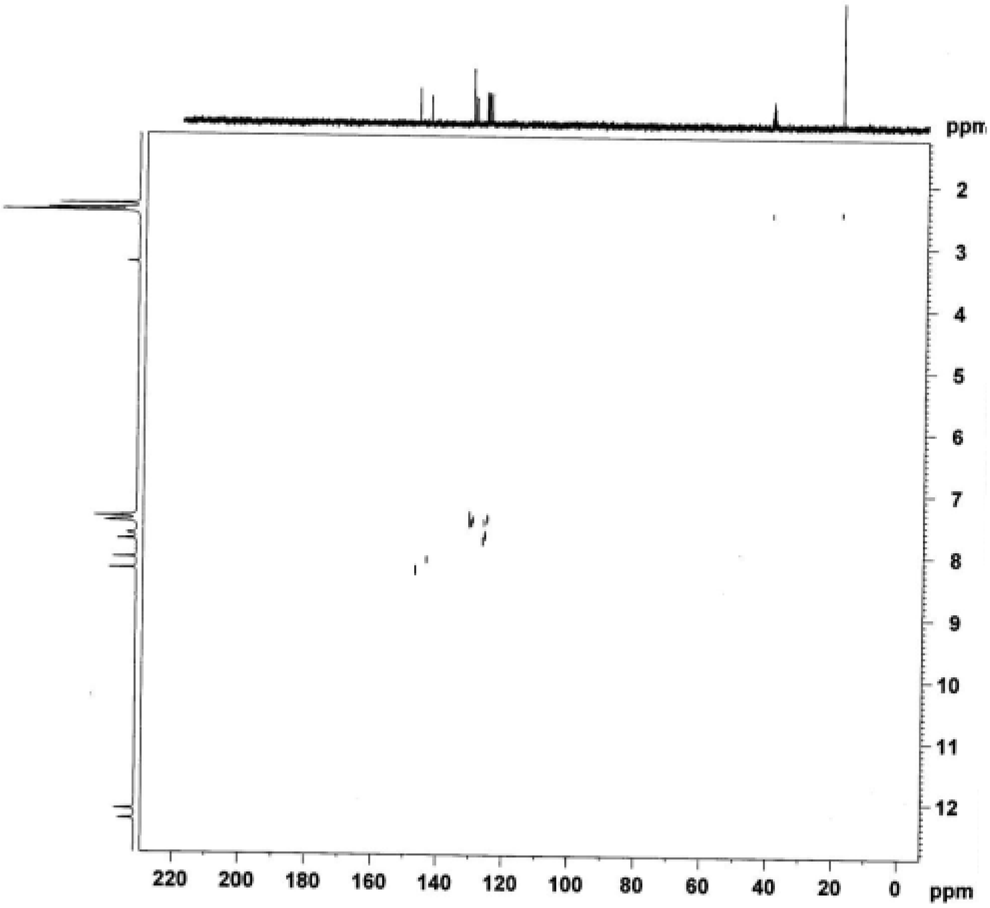

Figure **S34.** ^{1}H-^{13}C NMR spectrum of compound **5.**

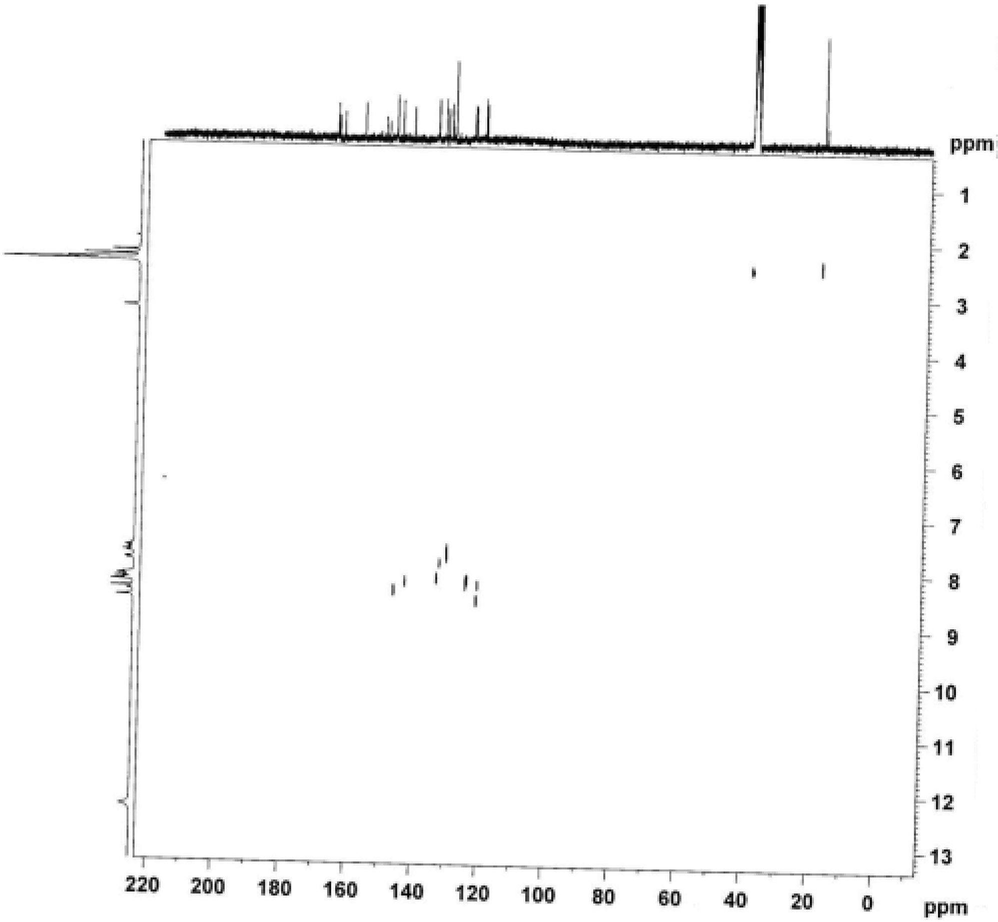

Figure S35. ^1H-^{13}C NMR spectrum of compound **6.**

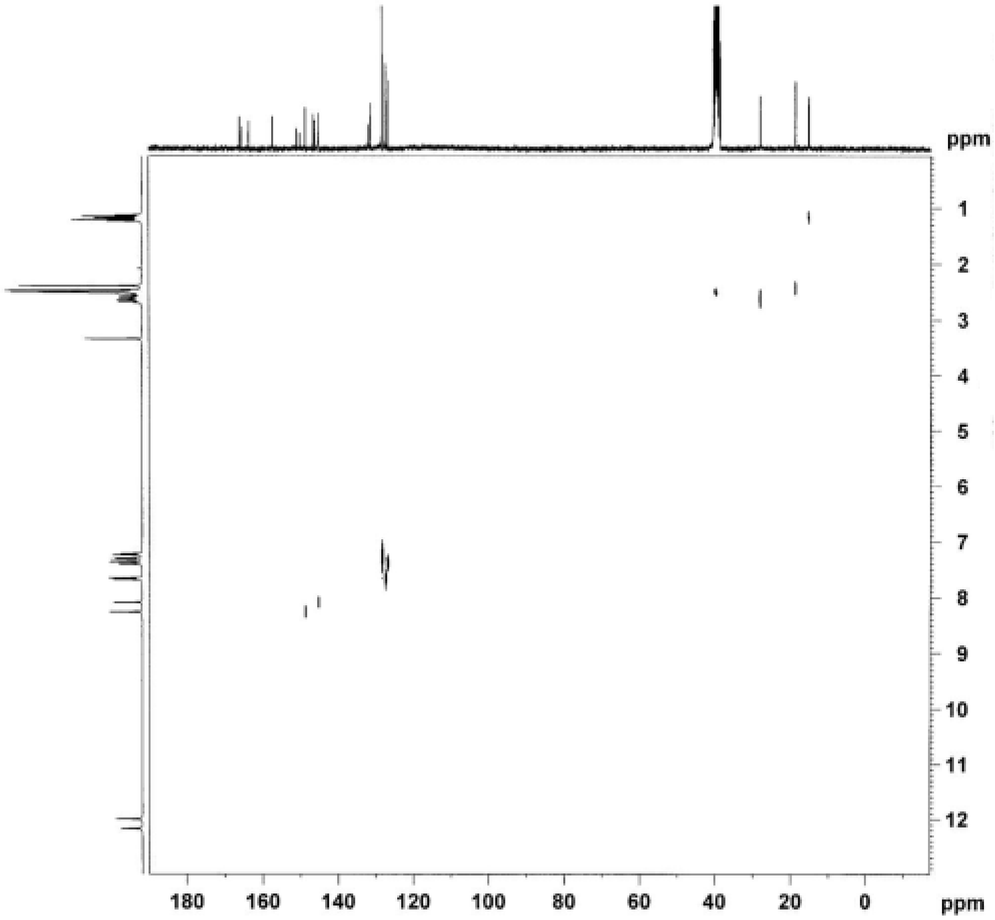

Figure S36. ^{1}H-^{13}C NMR spectrum of compound **7.**

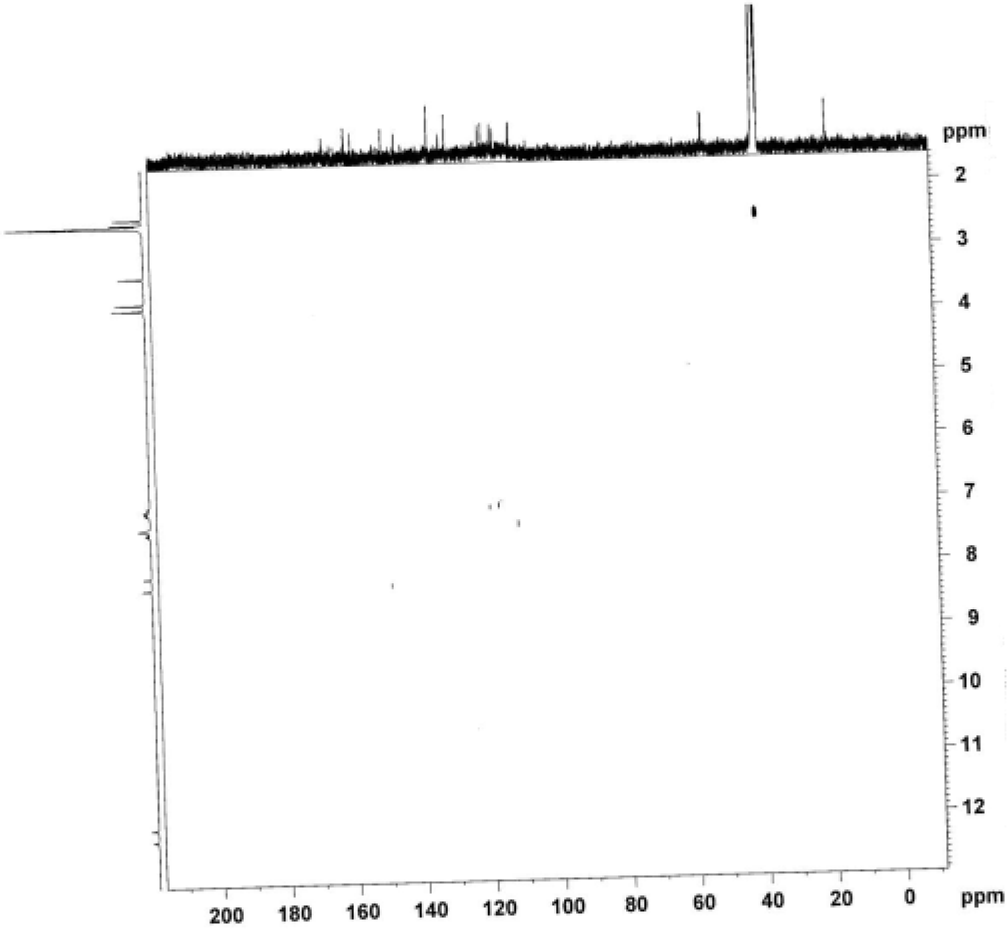

Figure S37. ^{1}H-^{13}C NMR spectrum of compound **8.**

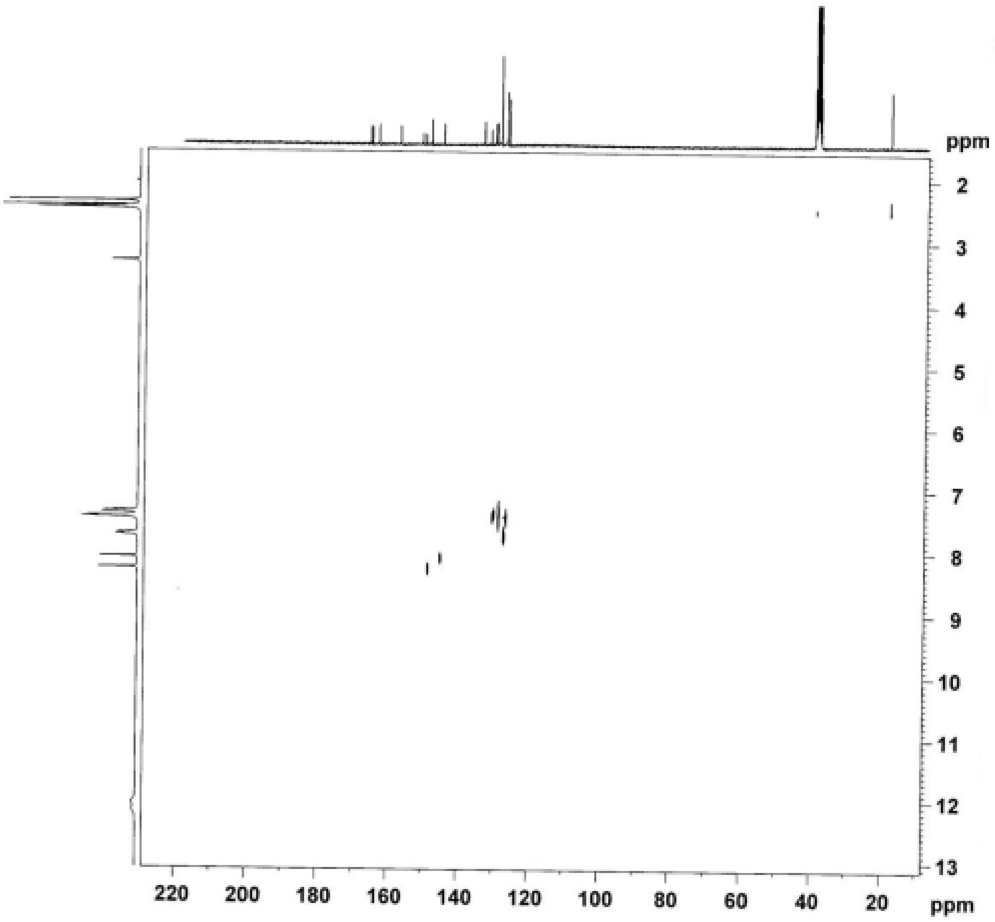

Figure S38. ^{1}H-^{13}C NMR spectrum of compound **9.**

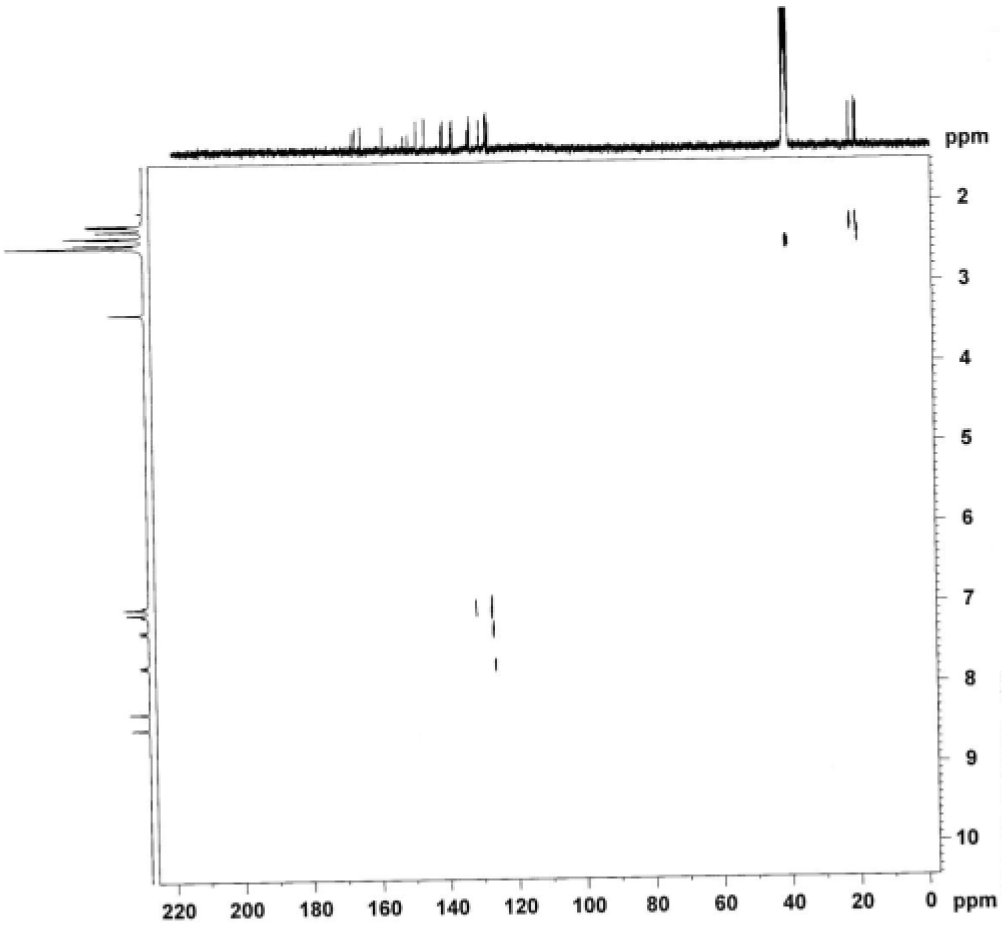

Figure S39. ^{1}H-^{13}C NMR spectrum of compound **10.**

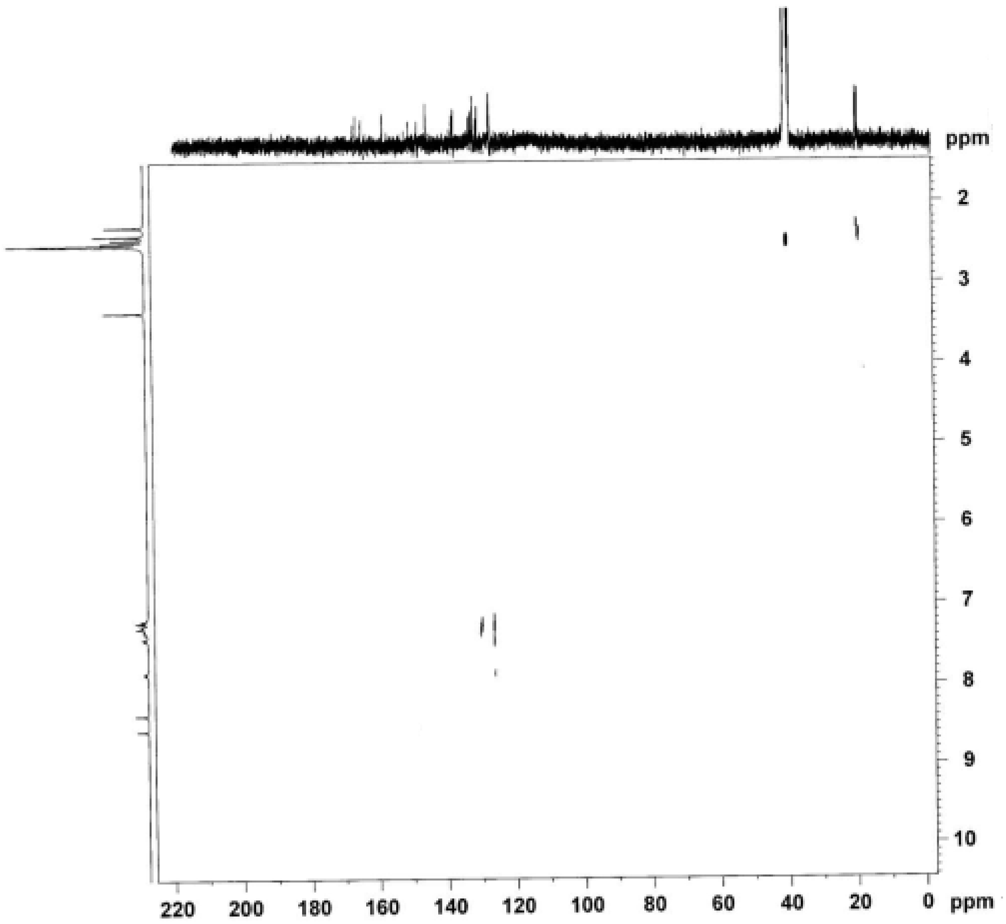

Figure S40. ^{1}H-^{13}C NMR spectrum of compound **11.**

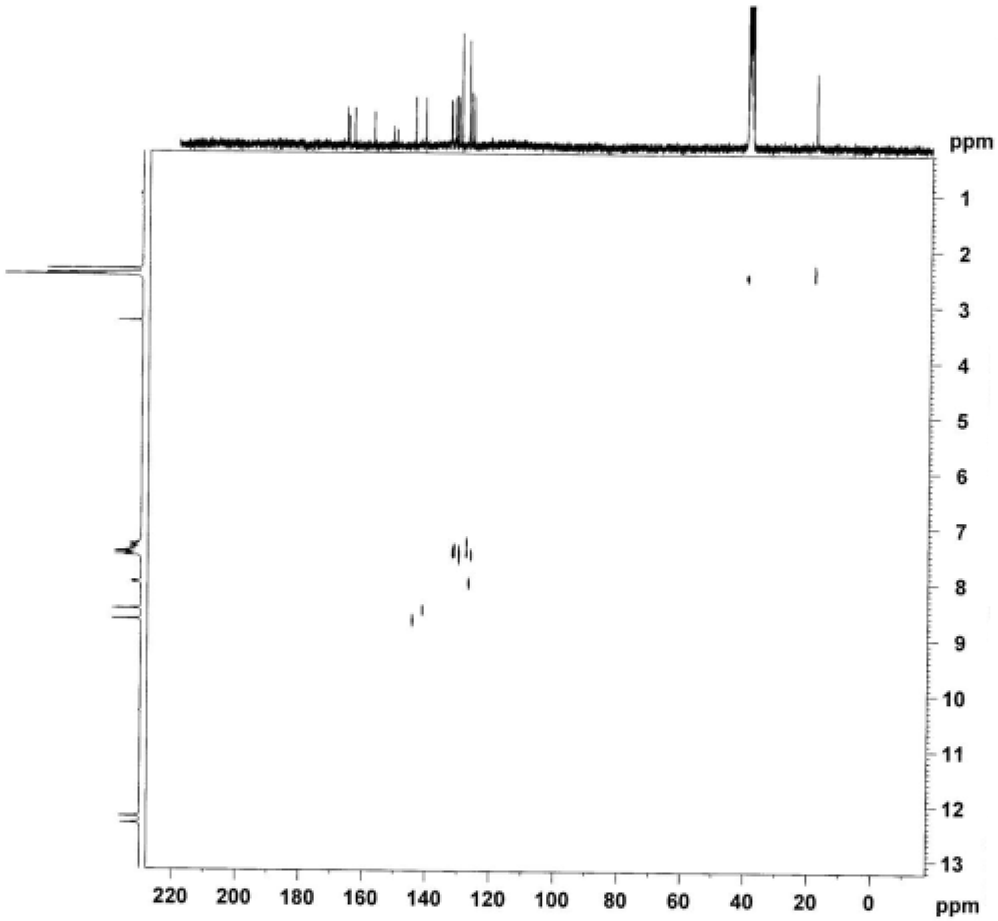

Supplement: Supplementary file 1 [file molecules-25-00088-s001.pdf]
